# Ultrahigh oxygen evolution reaction activity in Au doped co-based nanosheets†

**DOI:** 10.1039/d1ra09094a

**Published:** 2022-02-22

**Authors:** Chao Cai, Shaobo Han, Xiaotao Zhang, Jingxia Yu, Xia Xiang, Jack Yang, Liang Qiao, Xiaotao Zu, Yuanzheng Chen, Sean Li

**Affiliations:** Yangtze Delta Region Institute (Huzhou), University of Electronic Science and Technology of China Huzhou 313001 China xtzu@uestc.edu.cn; School of Physical Science and Technology, Key Laboratory of Advanced Technology of Materials (Ministry of Education of China), Southwest Jiaotong University Chengdu Sichuan 610031 China cyz@swjtu.edu.cn; School of Materials Science and Engineering, The University of New South Wales Sydney 2052 Australia sean.li@unsw.edu.au; Institute of Fundamental and Frontier Sciences, University of Electronic Science and Technology of China Chengdu 610054 P. R. China

## Abstract

Oxygen evolution reaction (OER) has attracted enormous interest as a key process for water electrolysis over the past years. The advance of this process relies on an effective catalyst. Herein, we employed single-atom Au doped Co-based nanosheets (NSs) to theoretically and experimentally evaluate the OER activity and also the interaction between Co and Au. We reveal that Au–Co(OH)_2_ NSs achieved a low overpotential of 0.26 V at 10 mA cm^−2^. This extraordinary phenomenon presents an overall superior performance greater than state-of-the-art Co-based catalysts in a sequence of α-Co(OH)_2_ < Co_3_O_4_ < CoOOH < Au–Co(OH)_2_. With *ab initio* calculations and analysis in the specific Au–Co(OH)_2_ configuration, we reveal that OER on highly active Au–Co(OH)_2_ originates from lattice oxygen, which is different from the conventional adsorbate evolution scheme. Explicitly, the configuration of Au–Co(OH)_2_ gives rise to oxygen non-bonding (O_NB_) states and oxygen holes, allowing direct O–O bond formation by a couple of oxidized oxygen with oxygen holes, offering a high OER activity. This study provides new insights for elucidating the origins of activity and synthesizing efficient OER electrocatalysts.

## Abstract 2

Manipulating Co oxidation by loading Au atoms can substantially enhance the OER activity of those activated Co ions.

## Graphical abstract

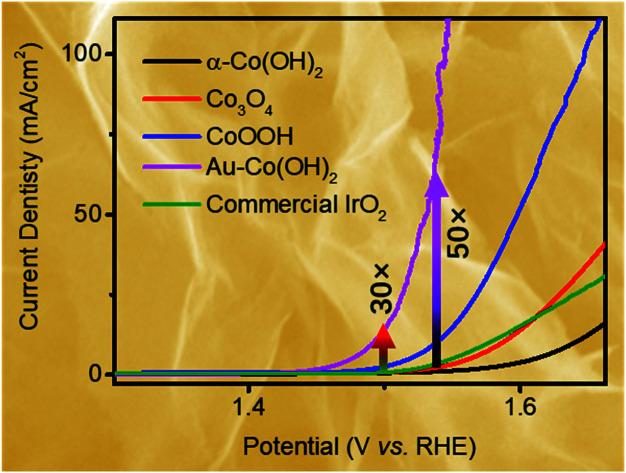

## Introduction

The hydrogen economy is a sunrise industry that will provide a solution for the energy crisis and greenhouse emissions as well as stimulate the rapid growth of the economy.^1,2^ The core of research and development in the hydrogen economy is to search for cost-effective and ultra-efficient electrocatalysts for oxygen evolution reaction (OER) to realize high throughput hydrogen production on an industrial scale.^3^ Although the noble metal-based OER catalysts including Ru and Ir oxides exhibit high OER kinetics with a current density of 10 mA cm^−2^,^4^ high-cost and limited resources make these materials would not be sought after by the industry. Instead, earth-abundant metal-based nanomaterials have been refocused as alternatives to the noble metal-based OER catalysts. These include the first-row transition-metal (3d) oxides,^5,6^ nitrides,^7^ sulfides,^8^ and hydroxides.^9^

Cobalt (Co)-based nanomaterials are the typical one because of their natural abundance and good OER performance.^10,11^ In particular, the multivalent nature of Co cations enables additional electrooxidation occurring in Co-based OER catalysts at high reaction potential [usually >1.23 V *versus* reversible hydrogen electrode (*vs.* RHE)]. The current efforts in developing efficient OER catalysts is to create the accelerated intrinsic active sites through manipulating the reaction conditions. Importantly, the formation of high-valance Co cations in the OER process can be facilitated through doping technology.^12–14^ For example, CoFe (oxy)hydroxides nanosheets (NSs) have high OER activity induced by the synergistic effects between Fe and Co, abundant oxygen and metal vacancies.^15^ It is reported that Au nanostructures can effectively enhance OER activity by positioning Au in a specific site to localize Au–Co interactions.^16^ Although many efforts have been put in to study the mechanism of Co-based materials for OER,^11,17–20^ as emerging hybrid electrocatalysts, the mechanism of the improved Co oxidation process is unclear. This is because the oxidation process of Co-based materials in the oxygen evolution reaction is too fast to be determined. It obstructs understanding the large activity gap between (oxy)hydroxides and precursors, impeding the development and rational design of high activity 3d metal-based electrocatalysts. Therefore, developing Co-based materials with a controllable oxidation state would be the first step to advance the highly active OER electrocatalysts.

Herein, we deposited ultrathin NSs of Co(OH)_2_-based catalysts on the surface of a highly electronegative Au single-atoms. By comparing non-modified Co-based NSs (*e.g.*, Co(OH)_2_, Co_3_O_4_, CoOOH), we were able to explore their OER activity and interaction between Co and Au. We found that the OER activities of those NSs are IrO_2_ < α-Co(OH)_2_ < Co_3_O_4_ < CoOOH < Au–Co(OH)_2_ based on the geometric current density. The OER activity of Au–Co(OH)_2_ NSs is 30-fold of commercial IrO_2_. The surface chemistry of this particular material was analyzed using X-ray photoelectron spectroscopy (XPS). *Ex situ* scanning transmission electron microscopy (STEM) was used to characterize the preferential oxidation of Co atoms, which was coordinated with Au atoms, revealing the high structural stability of Au–Co(OH)_2_ NSs in an oxidation environment. Further theoretical calculations demonstrate that Au can improve the adsorption/desorption efficiency around the active Co sites during OER. It is believed that this is related to the decrease of *OH/*O free energy. The high OER activity of Au–Co(OH)_2_ NSs originates from the high intrinsic activity of Co as well as the strong synergistic effect between Au and Co, and the large efficacious active surface area.

## Results and discussions

### Synthesis and structural characterization of ultrathin Co-based NSs

**Scheme 1:**
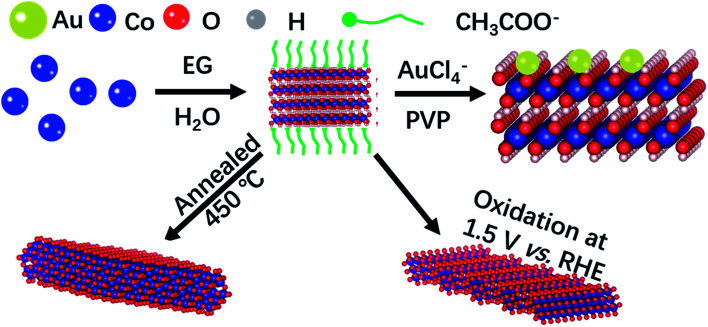
Growth procedure of ultrathin Co-based NSs. Co-Based NSs show high stability under operando conditions.

The ultrathin two-dimension feature of NSs ensures high processability at the atomic scale, such as surficial and interfacial modification. Here we demonstrate that the Co-based NSs with modified electronic configurations exhibit superior OER activity. The growth procedure of Co-based NSs is schematically illustrated in Scheme 1. α-Co(OH)_2_ crystals are assembled to form NSs with CH_3_COO^−^ adsorption, agreeing well with the reported Co-based NSs in ethylene glycol (EG).^10,21^ The manipulation of the oxidation state of Co was realized by processing α-Co(OH)_2_ NSs under operando conditions, forming Co_3_O_4_, CoOOH, and Au–Co(OH)_2_ NSs. The scanning electron microscopy (SEM) morphologies demonstrate the highly stable microstructure of Co-based NSs under operando conditions (Fig. S1†). As shown in Fig. S2,† peaks in the X-ray diffraction (XRD) pattern at 10.5°, 33.5° and 59.5° correspond to (003), (012) and (110) of α-Co(OH)_2_, respectively. Rhombohedral CoOOH was synthesized using the electrochemical oxidizing methodology from α-Co(OH)_2_ NSs. Subsequently, annealing α-Co(OH)_2_ formed Co_3_O_4_ NSs as shown in SEM morphology (Fig. S1† and XRD pattern in Fig. S2†).

**Fig. 1:**
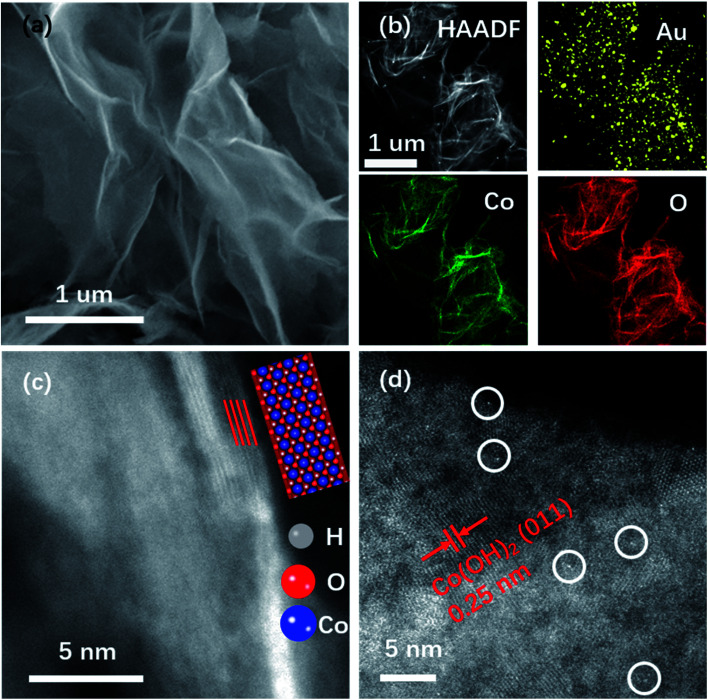
Structural characterization of Au–Co(OH)_2_. SEM image (a), EDS mapping (b), STEM image (c), and (d) HAADF image of Au–Co(OH)_2_. The newly formed Au–Co(OH)_2_ NSs maintain pure Co(OH)_2_ crystal features. NSs have four atomic layers with a thickness of 1.2 nm. Au attaches on NSs as single atoms, as indicated by white circles.

The double-Cs corrected high angle annular dark-field scanning transmission electron microscopy (HAADF-STEM) was used to determine Au on NSs. The SEM image in Fig. 1a shows the NS morphology of Au–Co(OH)_2_ and the elemental distribution with Au homogeneously distributed on Co(OH)_2_ NSs was mapped using Energy Dispersive Spectra (EDS) as shown in Fig. 1b. The HAADF-STEM image demonstrates that the NSs are composed of four layers with a total thickness of about 1.2 nm (Fig. 1c), agreeing well with the reported Co/Co–O NSs.^21^ In the HAADF image (Fig. 1d), the bright dots correspond to Au atoms while the others are Co atoms because of the higher mass of Au atoms. It shows that Au atoms are isolated and attached to the surface of Co(OH)_2_.

### Electrochemical characterization of Co-based NSs

**Fig. 2:**
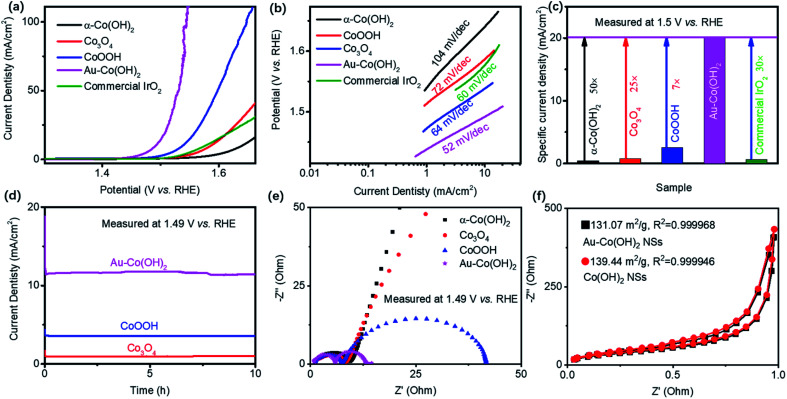
Electrochemical properties of the series of Co-based NSs in 1 M KOH. Polarization curves (a), Tafel plots (b), a collection of specific current destiny at 1.5 V *vs.* RHE (c), accelerating degradation (d), Nyquist plots (e), and N_2_ adsorption/desorption isothermal (f) of as-prepared Co-based NSs. The loaded Au on Co(OH)_2_ can highly promote the OER activity (a–c). The data in (c) is normalized by the geometric area of the working electrode. Co-based NSs show high stability for OER in alkaline solution (d). Au–Co(OH)_2_ NSs have the highest mass transport efficiency (e) among those NSs.

The OER performance of Co-based NSs was evaluated with 1 M KOH (Fig. 2). The current density was counted on the geometry of the working electrode. Fig. 2a shows the polarization curves of Co-based NSs. For comparison, the commercial IrO_2_ presents a specific current density of 10 mA cm^−2^ at an overpotential of 320 mV (1.55 V *vs.* RHE), which is similar to the reported data.^22,23^ The OER activity of Co-based NSs shows a sequence of Au–Co(OH)_2_ (260 mV) > CoOOH (308 mV) > commercial IrO_2_ (320 mV) > Co_3_O_4_ (357 mV) > α-Co(OH)_2_ (410 mV) with a benchmark of the overpotential in a specific current density of 10 mA cm^−2^. In addition, it is noted that the achieved OER activity of Au–Co(OH)_2_ is higher than that of the state-of-the-art Au/Co-based catalysts (Table S1†) including CoSe_2_,^24^ NiCo–A (A = P, Se, O)^6^ and Au–CoSe_2_.^25^ Tafel plots of these samples summarized in Fig. 2b demonstrate that Au–Co(OH)_2_ NSs possess the highest charge transport efficiency around active sites with a slope of 52 mV dec^−1^, which is smaller than that of Co_3_O_4_ NSs (64 mV dec^−1^), commercial IrO_2_ (60 mV dec^−1^), α-Co(OH)_2_ NSs (104 mV dec^−1^), and CoOOH NSs (74 mV dec^−1^). The specific current density of Co-based NSs at 1.5 V *vs.* RHE is plotted in Fig. 2c. Au–Co(OH)_2_ NSs exhibit the highest OER activity, which is 30-fold higher than that of the commercial IrO_2_. Besides, the mass activity (MA) and turnover frequency (TOF) are calculated on basis of Fig. 2c.^26,27^ MA of Au–Co(OH)_2_ is 177 A g^−1^, which is much higher than 3.89 A g^−1^, 7 A g^−1^, 22.56 A g^−1^, and 5.66 A g^−1^ of Co(OH)_2_, Co_3_O_4_, CoOOH, and IrO_2_, respectively. The TOF of Au–Co(OH)_2_ is 27 s^−1^, which is much higher than 0.6 s^−1^, 1.07 s^−1^, 3.45 s^−1^, and 0.87 s^−1^ of Co(OH)_2_, Co_3_O_4_, CoOOH, and IrO_2_, respectively. These results demonstrate the high atomic utilization of Au–Co(OH)_2_ even better than that in IrO_2_. Fig. 2d shows the accelerating degradation of Co-based NSs at 1.49 V *vs.* RHE, showing a transformation from Co(OH)_2_ to CoOOH during the OER processing (Fig. S3†). It demonstrates that the stability of electrocatalysts may originate from CoO_*x*_(OH)_*y*_ (*e.g.* CoOOH).

It is believed that the superior OER performance of Co-based NSs is related to the enhancement of electrical conductivity, which facilitates the relevant charge transfer between the support and catalysts.^28,29^ In this case, we used electrochemical impedance spectrometry (EIS) to characterize the electrode kinetics with different catalyst loading (Fig. 2e). Au–Co(OH)_2_ NSs demonstrate their highest charge transfer efficiency in OER at 1.49 V *vs.* RHE. To clarify that this phenomenon was not contributed by active sites number variations, we conducted the N_2_ adsorption/desorption isotherm test on Co-based NSs. Co(OH)_2_ NSs showed a higher surface area of 139.44 m^2^ g^−1^ than 131.07 m^2^ g^−1^ of Au–Co(OH)_2_ NSs (Fig. 2f). Even with the low active surface area of Au–Co(OH)_2_ NSs, which is 10-fold higher than that of pristine Co-based NSs. These results demonstrate that Au atoms on Co(OH)_2_ can efficiently increase the intrinsic activity of bonded Au–Co for OER. Therefore, we believe such exotic OER performance is associated with the highly efficient charge transfer and high intrinsic catalytic activity.

### The oxidation state of Co-based NSs

**Fig. 3:**
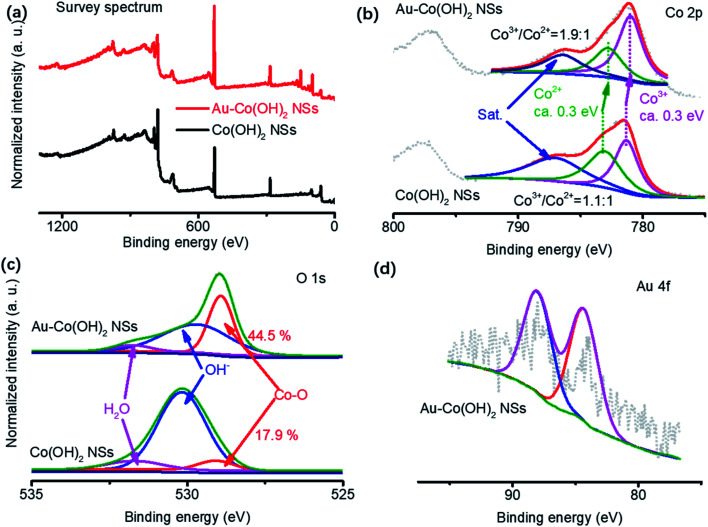
Surface chemistry characterization of Co(OH)_2_-based NSs. Survey (a), Co 2p spectrum (b), O 1s (c), and Au 4f spectrum (d). The extra Au can promote Co valence (c), and thus promotes the activity of high valence Co in OER.

High-valance Co (CoO_*x*_(OH)_*y*_) are usually considered the active sites for OER (Co^3+^/Co^4+^ group peaks located at 1.24–1.54 V *vs.* RHE).^16^ The OER activity of Co-based materials is highly sensitive to the oxygen vacancies surrounding Co cations. This is because the two electrons in the neighboring oxygen vacancies can be delocalized around Co^3+^/Co^4+^, ensuring that the low-coordinated Co sites are more active in adsorbing water molecules or active intermediate during OER.^30^ In our experiments, therefore, the core factors to analyze OER mechanisms are to clarify the oxidation states of species in Co(OH)_2_ NSs and Au–Co(OH)_2_ NSs. XPS was used to reveal the valence of cations and surface chemistry in NSs (Fig. 3). Spectra of Co, O, and Au as well as their deconvolution patterns are shown in Fig. 3a and b. It shows that the peaks at 781. 4 eV and 783.2 eV are assigned to Co^3+^ and Co^2+^ respectively.^10,31^ Co 2p_3/2_ has a red shift of 0.3 eV with the coated Au on Co(OH)_2_ NSs. The increased Co^3+^/Co^2+^ ratio in Au–Co(OH)_2_ NSs demonstrates that Au coating increases the Co valence in Co(OH)_2_, which was resulting from the low coordinated Co–O bonding because of the appearance of Au.^12^ Further study on O 1s shown in Fig. 3c indicates that spectra of O 1s can be deconvoluted into three peaks of Co–O bonding (529.1 eV), OH^−^ in Co(OH)_2_ (530.1 eV), and adsorbed H_2_O (531.8 eV).^32^ The higher ratio Co–O peak supports the notion that Co valence in Au–Co(OH)_2_ NSs is increased by Au coating. Fig. 3d shows the Au 4f peak, where two peaks located at 84.0 eV and 88.2 eV are corresponding to Au 4f_5/2_ and 4f_7/2_, respectively.^12^

**Fig. 4:**
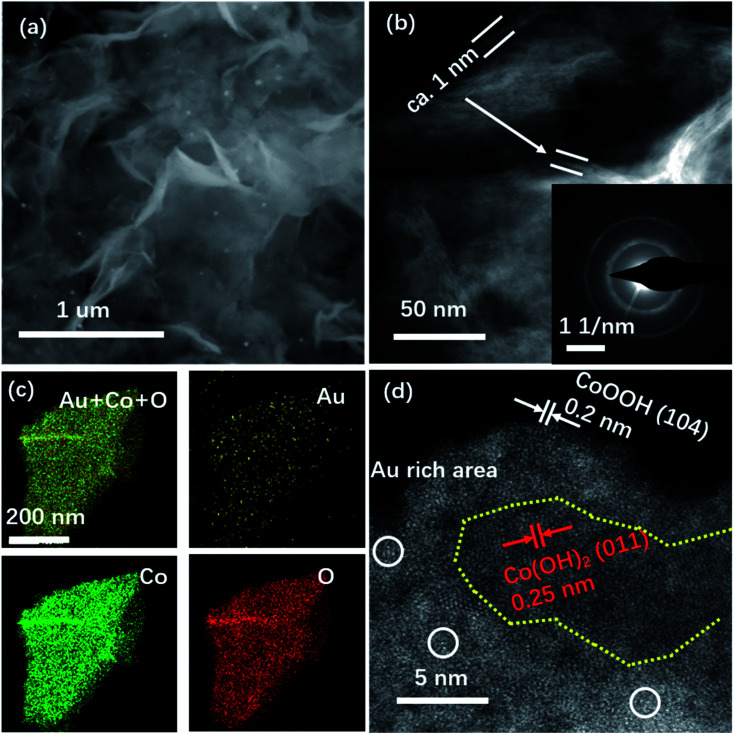
Structural characterization of Au–Co(OH)_2_ NSs after use. SEM image (a), HAADF image (b) (inset is SAED pattern), EDS mapping (c), and high magnification HAADF image of Au–Co(OH)_2_ NSs after use (d). Au–CoO_*x*_(OH)_*y*_ NSs have high structural stability in OER (a and b). Co^2+^ ions around the Au atoms are preferentially activated and changed into Co^3+^, because of the accelerated OER process around Au–Co couples. Yellow dots mark the pure Co(OH)_2_ in NSs after use.

In general, the OER catalysts with high activity are usually accompanied by structural instability related to oxidation. In this work, we conducted *ex situ* STEM experiments, XPS, and XRD to characterize the stability of Au–Co(OH)_2_ NSs in the accelerating degradation process as shown in Fig. 4, S4 and S5.† XPS analysis demonstrates a very limited change in the ratio of Co^2+^/Co^3+^ (1.8 : 1) with the accelerating degradation process, which is similar to the value of 1.9 : 1 for the as-prepared Au–Co(OH)_2_ NSs. It suggests that Au dominated the selective electro-oxidation of Co in OER. The STEM image in Fig. 4b shows that the thickness of Au–Co(OH)_2_ NSs is about 1 nm, demonstrating the chemical stability of Au–Co(OH)_2_ NSs. Fig. 4c shows the EDS mapping of the individual elements in Au–Co(OH)_2_ NSs after the stability characterization. As shown in Fig. 4d, Au atoms still maintain the isolated feature of CoO_*x*_(OH)_*y*_. The high-resolution HAADF STEM image exhibits the co-existence of CoOOH and Co(OH)_2_ (Fig. 4d), indicating that only a part of Co^2+^ cations in NSs adapt further electrooxidation during OER. This finding is supported by the *ex situ* XRD characterization (Fig. S5†), where it shows the co-existence of CoOOH and Co(OH)_2_ phase. The individual Au atoms are surrounded by *in situ* formed CoOOH (Fig. 4d). Moreover, compared to the oxidation barriers of Co^2+^ in Co(OH)_2_ and Au–Co(OH)_2_, we found that Co^2+^ in Au–Co(OH)_2_ is easier to be further oxidized into high valence Co^3+^ (Fig. S8 and Table S2†). These results show that Co atoms near Au are preferentially oxidized in OER and Au–CoOOH is responsible for the high OER activity (Fig. 2).

### Lattice-oxygen oxidation mechanism on Au–Co(OH)_2_

In general, the OER activity of Co-based materials is sensitive to the species loading amount. To clarify this, we synthesize samples with different Au loading amounts on Co(OH)_2_ NSs for OER. Fig. S6† shows the polarization curves and Tafel plots of Au–Co(OH)_2_ NSs. NSs with different Au doping levels show similar onset potential (1.43 V *vs.* RHE), demonstrating that the existence of Au in Co(OH)_2_ NSs to form Au–CoOOH is responsible for the high OER activity. Such a phenomenon may be attributed to the electron affinity difference between Au (223 kJ mol^−1^) and Co (64 kJ mol^−1^), where the higher electron affinity of Au enables the high concentration of negatively charged species (OH^−^, OOH^−^, and O^2−^) surrounding Au near Co cations. In this case, the improved activity of Au–Co(OH)_2_ NSs is dealt with the change of OH^−^ species around active sites on the surface, where it shows a difference between the adsorption free energy (X–OH) and the reduced O (X–O).^33^ Meanwhile, because of the electron affinity difference and activated Au with a low excited barrier, the addition of Au may enhance the efficiency of the reversible absorption/adsorption process.^34^ This procedure can facilitate the formation of adsorbed OH^−^ and reduced O intermediates on the Co^3+^ closed to the oxygen vacancies (agreeing well with Fig. S4†), enhancing the oxidation process from Co^3+^ to Co^4+^. This is also verified by the cyclic voltammetry method (Fig. S7†), where the integrated area of oxidation peak for the processes from Co^2+^ to Co^3+^ (1.1 V *vs.* RHE)^35^ and Co^3+^ to Co^4+^ (1.4 V *vs.* RHE) is increased 20-fold with individual Au atom deposition. However, further increasing the Au use in catalysts, the OER activity of Au–Co(OH)_2_ NSs decreases. It is believed that the excessive substitution is unfavorable to refill the oxygen vacancies through the lattice-oxygen oxidation mechanism (LOM).^36^ Consequently, Au excessive substitution in Au–Co(OH)_2_ would not only lower the overall activity but also lead to severe degradation of catalyst because of the significant loss of oxygen. Therefore, optimizing the Au doping level in Au–Co(OH)_2_ NSs can efficiently enhance the catalytic activity in the OER processing.

**Fig. 5:**
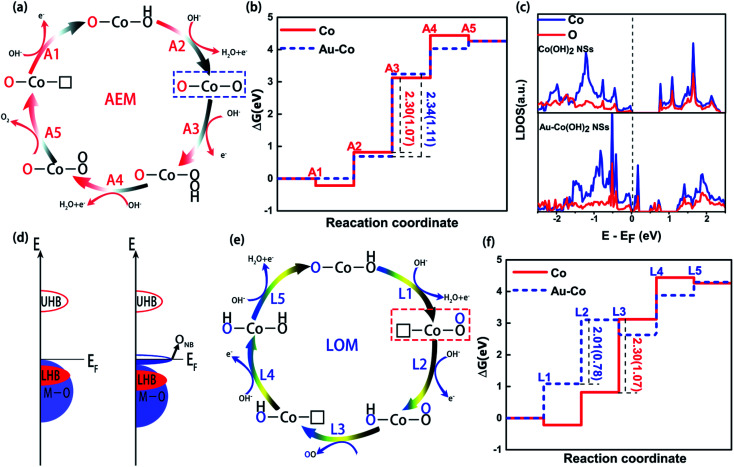
(a) The reaction pathways of OER for Au–Co(OH)_2_ in adsorbate evolution mechanism (AEM); (b) the free energy profiles of each reaction stage for Au–Co(OH)_2_ (denoted as Au–Co) and the pristine Co(OH)_2_ (denoted as Co) in AEM, and the Δ*G*_MAX_ are labeled and the overpotential value is marked in parentheses; (c) the projected DOS for the Au–Co(OH)_2_ in comparison with that of Co(OH)_2_; (d) the schematic energy bands for the Au–Co(OH)_2_ and Co(OH)_2_, in which the antibonding band is split into one empty upper-Hubbard band (UHB) and one filled lower-Hubbard band (LHB). The oxygen non-bonding state (O_NB_) occurs above the Fermi level in the energy bands of Au–Co(OH)_2_; (e) the reaction pathways of OER in lattice oxygen reaction (LOM), in which the key step difference from AEM is labeled by the dotted line; (f) the free energy profiles of each reaction stage for Au–Co(OH)_2_ and Co(OH)_2_ in LOM.

In order to understand the reaction mechanism of the OER process of Au–Co(OH)_2_ nanosheets, we used the traditional adsorbate evolution mechanism to study the Gibbs free energy of possible adsorbates on their surfaces. In general, the basic OER process involves four fundamental electrochemical processes and one of the following desorption processes, as shown in Fig. 5a and S9.† The computed Δ*G* has no obvious difference between the Au–Co(OH)_2_ and Co(OH)_2_ (Fig. 5b). Their theoretical overpotential, which is equal to |Δ*G*_MAX_ − 1.23|, is nearly unchanged (1.11 eV for the Au–Co(OH)_2_, 1.01 eV for Co(OH)_2_). It demonstrates that traditional AEM cannot explain the observed high catalytic performance in the OER for Au–Co(OH)_2_s.

Recent studies on OER demonstrated that the doping of Co(OH)_2_ by heterogeneous atoms can oxidize oxygen atoms in lattice to generate oxygen with LOM. In this mechanism, the oxygen generated in the OER process is not only originating from the decomposition of water molecules, but also the oxidation of lattice oxygen atoms and adsorbed oxygen atoms. It is believed that this process improves the OER performance of the materials significantly. Considering the strong Coulomb effect of the d–d orbits between the heterogeneous atoms and the weak bonding formed between Au and Co, a similar ONB state is formed between Au and Co(OH)_2_. In this case, Au and Co(OH)_2_ would adsorb some unbonded oxygen. Therefore, it is inevitable that LOM states are formed on the surfaces of Au and Co(OH)_2_. In Au–Co(OH)_2_, as shown in Fig. 5c and S10,† the electric charge of Au along its *C*-axis region decreases, and subsequently transfers to the coordinated O atoms (Au–O bond direction). This forms the unoccupied ONB state above the Fermi level (*E*_F_). Moreover, the experiment indicates that the doped Au leads to the oxygen holes formation in Au–Co(OH)_2_. These experimental results reveal that the oxidized oxygen nearby oxygen holes in Au–Co(OH)_2_ is preferentially transformed into O–O. Herein, we proposed the likely reaction schemes of LOM for Au–Co(OH)_2_ involving the oxidation of lattice oxygen (Fig. 5e and S11†), though other possibilities may exist. This scheme mainly consists of two key chemical steps (L1 and L3), which first creates an O–O bond and subsequently an oxygen molecule from lattice oxygen sites *via* the formation of oxygen vacancies. As shown in Fig. 5f, Δ*G* calculations show that the overpotential decreases from 1.07 eV in Co(OH)_2_ to 0.78 eV in Au–Co(OH)_2_, suggesting that the OER of Au–Co(OH)_2_ along the LOM pathway is more thermodynamically favorable. It enables better performance in accordance with the experiment. Compared to AEM, the LOM mechanism can well explain the experimental observation in the highly efficient electrochemical performance of Au–Co(OH)_2_, which is related to the creation of O_NB_ states and oxygen holes of the specific Au–Co(OH)_2_ configuration.

## Conclusions

In this work, we synthesized Co-based NSs as OER catalysts. These Co-based NSs show a controllable oxidation state, leading to the OER activity change. Au–Co(OH)_2_ shows the best OER activity, which is 30-fold higher than the commercial IrO_2_. The existence of Au resulted in the OER activity adjustment by accelerating the oxidation process of high valence Co. This facilitates the adsorption/desorption efficiency on the activated Co center, enhancing the effective and active surface areas. The synergistic effect between Au and Co is highly sensitive to the d orbits of Au atoms, accelerating the O_NB_ formation and OER activity. The excited barriers in Au–Co(OH)_2_ are lowered, accelerating the oxidation process of Co during OER. The high intrinsic activity of Co is further enhanced by the individual Au atoms.

## Experimental

### Chemicals

Co(acac)_3_ (>95%), ethylene glycol (EG) (98%), KOH (>99.999%), ethanol (>97%), HAuCl_4_·4H_2_O (metal tracing, >42%), and isopropanol (>98%) were purchased from Aladdin. 5 wt% Nafion was purchased from Sigma.

### Preparation of α-Co(OH)_2_ NSs

In a typical synthesis of α-Co(OH)_2_ NSs, 200 mg of cobalt acetylacetonate (Co(acac)_3_) was dissolved in 40 mL of EG. The EG solution containing Co(acac)_3_ was mixed with 8 mL deionized water, heated at 160 °C for 48 h, and then cooled down to room temperature. The product was washed with a mixture of ethanol and deionized water several times, collected by centrifugation (10 000 rpm, 5 min), and then dried in a vacuum oven at 70 °C, overnight.

### Preparation of CoOOH NSs

20 mg fresh α-Co(OH)_2_ NSs were cast onto silicon support, and then a potential of 1.5 V *vs.* RHE was applied on the silicon support using chronoamperometry in 1 M KOH. After 10 h, the samples were sonicated and collected by centrifugation, washed with water and ethanol several times.

### Preparation of Co_3_O_4_ NSs

The as-prepared α-Co(OH)_2_ NSs were annealed at 400 °C for 5 min in the air (heating rate is *ca.* 10 °C min^−1^), and then cooled to room temperature. The black powders were obtained.

### Preparation of Au–Co(OH)_2_ NSs

According to the reports,^37^ α-Co(OH)_2_ NSs (40 mg) and required amounts of Au (2, 4, 10, 20, and 40 μL HAuCl_4_·4H_2_O (20 mg mL^−1^)) were dispersed in 10 mL ethanol under vigorous stirring, overnight. 1 mL NaBH_4_ solution (2 mg mL^−1^) was added to the solution dropwise. After stirring for 1 h, Au–Co(OH)_2_ NSs were collected and washed with water and ethanol, dried in a vacuum oven at 70 °C, overnight.

### Structure characterization

The morphology and microstructure characterizations of the as-prepared samples were investigated using scanning electron microscopy (SEM) (FEI, Helios Nanolab 600i, 2 kV), transmission electron microscopy (TEM) and selected area electron diffraction (SAED) (FEI, ETEM, G2, 200 kV), scanning transmission electron microscopy with an energy-dispersive X-ray spectroscopy attachment (STEM-EDS, FEI Titan Themis, 300 kV). X-ray diffraction (XRD) (Bruker ECO D8 power X-ray diffractometer with Cu Kα radiation) was utilized to determine the crystal structure of the samples.

### Electrochemical characterization

All experiments were carried out using the CHI760e in a three-electrode system. Rotate glassy disk carbon electrode (GCE) (3 mm) was the support of active materials to be used as the working electrode. Ag/AgCl (saturated with 4 M KCl) and a graphite rod were used as counter electrode and reference electrode, respectively. The electrolyte was 1 M KOH saturated with O_2_ (99.999)%. The working electrode for Co-based NSs was prepared by dropping 4 μL dispersion (catalyst ink (2 mg mL^−1^) with isopropanol/water (1 : 3) containing 10 μL 5 wt% Nafion solution) in a GCE. Cycling voltammetry (CV), linear sweep voltammetry (LSV), chronoamperometry and Tafel plot were measured at 298 K, 1600 rpm, and 5 mV s^−1^ on ALS-RRDE. The prepared working electrode was soaked in 1 M KOH solution for 2 h to form a Na^+^ Nafion before electrochemistry was conducted.

### Computational models and methods

Vienna *Ab initio* Simulation Package was used for all density functional theory (DFT) calculations. The projector-augmented plane-wave method was performed. Exchange–correlation functional was treated in the Perdew–Burke–Ernzerhof (PBE). Fully relaxed calculations were simulated with 2 × 2 × 1 supercells. We used a slab cut along the (001) direction of Co(OH)_2_ as a model for the terminated surface. Au atom was introduced afterward in the model and then was optimized to the lowest energy position. The constructed supercell was isolated with an 18 Å vacuum space in the *z*-direction. The Brillouin zones of all systems were sampled with gamma-centred grids. The 10 × 10 × 1 gamma *k*-point setups were used for all structure optimization. The force and energy convergence criteria were set to 0.02 eV Å^−1^ and 10^−5^ eV, respectively. The Gibbs free energy calculations considered the zero-point energy (ZPE) in AEM and LOM pathways.

## Conflicts of interest

There are no conflicts to declare.

## Supplementary Material

RA-012-D1RA09094A-s001

## References

[cit1] Kanan M. W., Nocera D. G. (2008). In situ formation of an oxygen-evolving catalyst in neutral water containing phosphate and Co^2+^. Science.

[cit2] Seitz L. C., Dickens C. F., Nishio K., Hikita Y., Montoya J., Doyle A., Kirk C., Vojvodic A., Hwang H. Y., Norskov J. K., Jaramillo T. F. (2016). A highly active and stable IrO_*x*_/SrIrO_3_ catalyst for the oxygen evolution reaction. Science.

[cit3] Pfisterer J. H. K., Liang Y., Schneider O., Bandarenka A. S. (2017). Direct instrumental identification of catalytically active surface sites. Nature.

[cit4] Lee Y., Suntivich J., May K. J., Perry E. E., Shao-Horn Y. (2012). Synthesis and Activities of Rutile IrO_2_ and RuO_2_ Nanoparticles for Oxygen Evolution in Acid and Alkaline Solutions. J. Phys. Chem. Lett..

[cit5] Zhou D., Wang S., Jia Y., Xiong X., Yang H., Liu S., Tang J., Zhang J., Liu D., Zheng L., Kuang Y., Sun X., Liu B. (2019). NiFe Hydroxide Lattice Tensile Strain: Enhancement of Adsorption of Oxygenated Intermediates for Efficient Water Oxidation Catalysis. Angew. Chem., Int. Ed..

[cit6] Fang Z., Peng L., Qian Y., Zhang X., Xie Y., Cha J. J., Yu G. (2018). Dual Tuning of Ni-Co-A (A = P, Se, O) Nanosheets by Anion Substitution and Holey Engineering for Efficient Hydrogen Evolution. J. Am. Chem. Soc..

[cit7] Zhang B., Xiao C., Xie S., Liang J., Chen X., Tang Y. (2016). Iron–Nickel Nitride Nanostructures *in Situ* Grown on Surface-Redox-Etching Nickel Foam: Efficient and Ultrasustainable Electrocatalysts for Overall Water Splitting. Chem. Mater..

[cit8] Wang H.-F., Tang C., Wang B., Li B.-Q., Zhang Q. (2017). Bifunctional Transition Metal Hydroxysulfides: Room-Temperature Sulfurization and Their Applications in Zn-Air Batteries. Adv. Mater..

[cit9] Huang J., Chen J., Yao T., He J., Jiang S., Sun Z., Liu Q., Cheng W., Hu F., Jiang Y., Pan Z., Wei S. (2015). CoOOH Nanosheets with High Mass Activity for Water Oxidation. Angew. Chem., Int. Ed..

[cit10] Sun Y., Gao S., Lei F., Liu J., Liang L., Xie Y. (2014). Atomically-thin non-layered cobalt oxide porous sheets for highly efficient oxygen-evolving electrocatalysts. Chem. Sci..

[cit11] Cai C., Wang M., Han S., Wang Q., Zhang Q., Zhu Y., Yang X., Wu D., Zu X., Sterbinsky G. E., Feng Z., Gu M. (2021). Ultrahigh Oxygen Evolution Reaction Activity Achieved Using Ir Single Atoms on Amorphous CoOx Nanosheets. ACS Catal..

[cit12] Zhuang Z., Sheng W., Yan Y. (2014). Synthesis of Monodisperse Au@Co_3_O_4_ Core-Shell Nanocrystals and Their Enhanced Catalytic Activity for Oxygen Evolution Reaction. Adv. Mater..

[cit13] Song F., Hu X. (2014). Ultrathin cobalt-manganese layered double hydroxide is an efficient oxygen evolution catalyst. J. Am. Chem. Soc..

[cit14] Fester J., Makoveev A., Grumelli D., Gutzler R., Sun Z., Rodriguez-Fernandez J., Kern K., Lauritsen J. V. (2018). The Structure of the Cobalt Oxide/Au Catalyst Interface in Electrochemical Water Splitting. Angew. Chem., Int. Ed..

[cit15] Wang Y., Zhang Y., Liu Z., Xie C., Feng S., Liu D., Shao M., Wang S. (2017). Layered Double Hydroxide Nanosheets with Multiple Vacancies Obtained by Dry Exfoliation as Highly Efficient Oxygen Evolution Electrocatalysts. Angew. Chem., Int. Ed..

[cit16] Yeo B. S., Bell A. T. (2011). Enhanced Activity of Gold-Supported Cobalt Oxide for the Electrochemical Evolution of Oxygen. J. Am. Chem. Soc..

[cit17] Sidhureddy B., Thiruppathi A. R., Chen A. (2017). Au nanoparticle incorporated Co(OH)(2) hybrid thin film with high electrocatalytic activity and stability for overall water splitting. J. Electroanal. Chem..

[cit18] Abu Sayeed M., Herd T., O'Mullane A. P. (2016). Direct electrochemical formation of nanostructured amorphous Co(OH)(2) on gold electrodes with enhanced activity for the oxygen evolution reaction. J. Mater. Chem. A.

[cit19] Frydendal R., Busch M., Halck N. B., Paoli E. A., Krtil P., Chorkendorff I., Rossmeisl J. (2015). Enhancing Activity for the Oxygen Evolution Reaction: The Beneficial Interaction of Gold with Manganese and Cobalt Oxides. Chemcatchem.

[cit20] Moysiadou A., Lee S., Hsu C.-S., Chen H. M., Hu X. (2020). Mechanism of Oxygen Evolution Catalyzed by Cobalt Oxyhydroxides: Cobalt Superoxide Species as a Key Intermediate and Dioxygen Release as a Rate-Determining Step. J. Am. Chem. Soc..

[cit21] Gao S., Lin Y., Jiao X., Sun Y., Luo Q., Zhang W., Li D., Yang J., Xie Y. (2016). Partially oxidized atomic cobalt layers for carbon dioxide electroreduction to liquid fuel. Nature.

[cit22] Cai C., Han S., Caiyang W., Zhong R., Tang Y., Lawrence M. J., Wang Q., Huang L., Liang Y., Gu M. (2018). Tracing the Origin of Visible Light Enhanced Oxygen Evolution Reaction. Adv. Mater. Interfaces.

[cit23] Cai C., Mi Y., Han S., Wang Q., Liu W., Wu X., Zheng Z., Xia X., Qiao L., Zhou W., Zu X. (2019). Engineering ordered dendrite-like nickel selenide as electrocatalyst. Electrochim. Acta.

[cit24] Kwak I. H., Im H. S., Jang D. M., Kim Y. W., Park K., Lim Y. R., Cha E. H., Park J. (2016). CoSe(2) and NiSe(2) Nanocrystals as Superior Bifunctional Catalysts for Electrochemical and Photoelectrochemical Water Splitting. ACS Appl. Mater. Interfaces.

[cit25] Zhao X., Gao P., Yan Y., Li X., Xing Y., Li H., Peng Z., Yang J., Zeng J. (2017). Gold atom-decorated CoSe2 nanobelts with engineered active sites for enhanced oxygen evolution. J. Mater. Chem. A.

[cit26] Wang J., Cai C., Wang Y., Yang X., Wu D., Zhu Y., Li M., Gu M., Shao M. (2021). Electrocatalytic Reduction of Nitrate to Ammonia on Low-Cost Ultrathin CoOx Nanosheets. ACS Catal..

[cit27] Cai C., Liu K., Zhu Y., Li P., Wang Q., Liu B., Chen S., Li H., Zhu L., Li H., Fu J., Chen Y., Pensa E., Hu J., Lu Y.-R., Chan T.-S., Cortes E., Liu M. (2021). Optimizing Hydrogen Binding on Ru Sites with RuCo Alloy Nanosheets for Efficient Alkaline Hydrogen Evolution. Angew. Chem., Int. Ed..

[cit28] Zhou H., Yu F., Sun J., He R., Chen S., Chu C. W., Ren Z. (2017). Highly active catalyst derived from a 3D foam of Fe(PO3)2/Ni2P for extremely efficient water oxidation. Proc. Natl. Acad. Sci. U. S. A..

[cit29] Zhang J., Zhang Q., Feng X. (2019). Support and Interface Effects in Water-Splitting Electrocatalysts. Adv. Mater..

[cit30] Bao J., Zhang X. D., Fan B., Zhang J. J., Zhou M., Yang W. L., Hu X., Wang H., Pan B. C., Xie Y. (2015). Ultrathin Spinel-Structured Nanosheets Rich in Oxygen Deficiencies for Enhanced Electrocatalytic Water Oxidation. Angew. Chem., Int. Ed..

[cit31] Liu Y., Jin Z., Li P., Tian X., Chen X., Xiao D. (2018). Boron- and Iron-Incorporated α-Co(OH)_2_ Ultrathin Nanosheets as an Efficient Oxygen Evolution Catalyst. ChemElectroChem.

[cit32] Zhang H., Zhang J., Li Y., Jiang H., Jiang H., Li C. (2019). Continuous oxygen vacancy engineering of the Co3O4 layer for an enhanced alkaline electrocatalytic hydrogen evolution reaction. J. Mater. Chem. A.

[cit33] Zhuang L., Ge L., Yang Y., Li M., Jia Y., Yao X., Zhu Z. (2017). Ultrathin Iron-Cobalt Oxide Nanosheets with Abundant Oxygen Vacancies for the Oxygen Evolution Reaction. Adv. Mater..

[cit34] Zhang J., Liu J., Xi L., Yu Y., Chen N., Sun S., Wang W., Lange K. M., Zhang B. (2018). Single-Atom Au/NiFe Layered Double Hydroxide Electrocatalyst: Probing the Origin of Activity for Oxygen Evolution Reaction. J. Am. Chem. Soc..

[cit35] Liang Y. Y., Wang H. L., Zhou J. G., Li Y. G., Wang J., Regier T., Dai H. J. (2012). Covalent Hybrid of Spinel Manganese-Cobalt Oxide and Graphene as Advanced Oxygen Reduction Electrocatalysts. J. Am. Chem. Soc..

[cit36] Huang Z.-F., Song J., Du Y., Xi S., Dou S., Nsanzimana J. M. V., Wang C., Xu Z. J., Wang X. (2019). Chemical and structural origin of lattice oxygen oxidation in Co–Zn oxyhydroxide oxygen evolution electrocatalysts. Nat. Energy.

[cit37] Shariq M., Majeric P., Friedrich B., Budic B., Jenko D., Dixit A. R., Rudolf R. (2017). Application of Gold(III) Acetate as a New Precursor for the Synthesis of Gold Nanoparticles in PEG Through Ultrasonic Spray Pyrolysis. J. Cluster Sci..

