# Supplementary material for: Ultrahigh oxygen evolution reaction activity in Au doped co-based nanosheets
Source: RSC Adv. 2022 Feb 22;12(10):6205–13. doi: 10.1039/d1ra09094a (PMC8982178; doi:10.1039/d1ra09094a)
Supplement: RA-012-D1RA09094A-s001 [file RA-012-D1RA09094A-s001.pdf]

Electronic Supplementary Material (ESI) for RSC Advances.
This journal is © The Royal Society of Chemistry 2022

Ultrahigh Oxygen Evolution Reaction Activity in Au Doped Co-based Nanosheets

Cai et al.

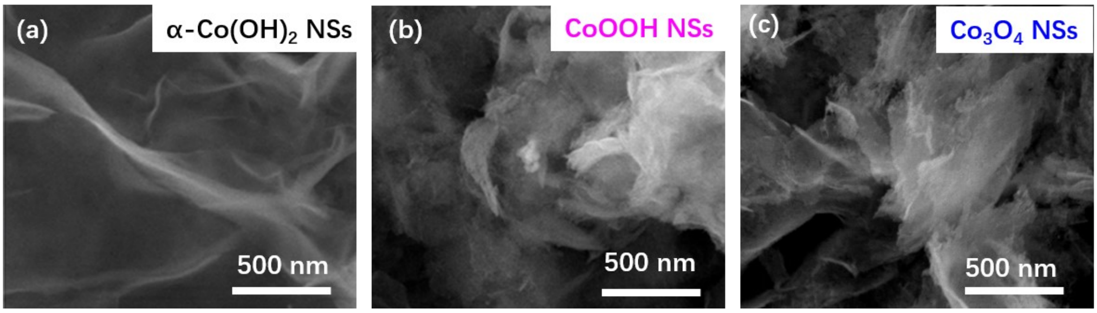

Figure S1. SEM images of Co-based NSs.

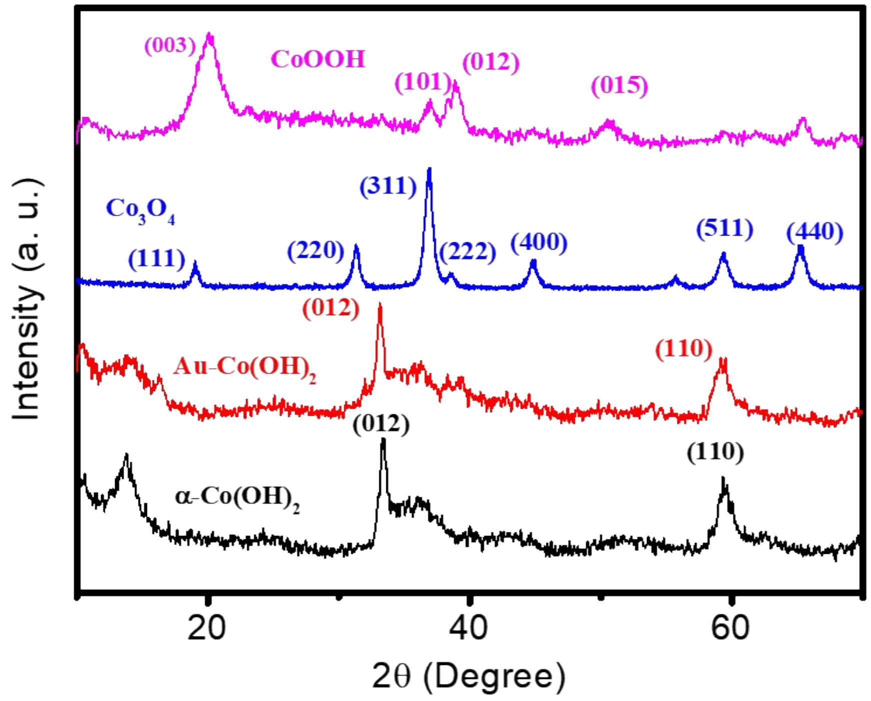

Figure S2. XRD pattern of Co-based NSs. As we can note that the diffraction peaks are identical with JCPDS No. 07-0169 (CoOOH). In detail, peaks located at 20.4°, 36.9°, 38.9°, 50.6°, and 65.3° are consistent with (003), (101), (012), (015), and (110) facet, respectively. The XRD pattern of Co_3O_4 NSs shows that peaks located at 19.0°, 31.3°, 36.9°, 44.8°, 55.6°, 59.4°, and 65.2° are contributed by (111), (220), (311), (400), (422), (511), and (440) facet, respectively (JCPDS No. 43-1003).

Table S1. Comparison of OER activity of some Au/Co-based electrocatalysts on glassy carbon electrode.

Sample	*electrolyte*	*Overpotential at 10 mA/cm²* *(mV)*	*Tafel slopes*	*Ref.*
Au/NiFe-LDH	1 M KOH	230	160	[1]
Pt/Fe-Au	1 M KOH	330	-	[2]
Ni_3S_2/Au-Graphene	1 M KOH	280	106	[3]
AuFe	1 M KOH	800	-	[4]
Au@$Ni_{12}P_5$	1 M KOH	340	49	[5]
Au@$CoFeO_x$	1 M KOH	328	-	[6]
Ni–Co–P	1 M KOH	270	76	[7]
$CoSe_2$	1 M KOH	430	--	[8]
NiCo-P	1 M KOH	330	--	[9]
Au-$CoSe_2$	1 M KOH	303	--	[10]
Au@Co_3O_4	1 M KOH	370	60	[11]
Au@Ni_3S_2	1 M KOH	280	70	[12]
CoOOH	1 M KOH	300	--	[13]
Au-Co(OH)$_2$ NSs	**1 M KOH**	**260**	**52**	**This work**

--. No specific value is mentioned in Ref.

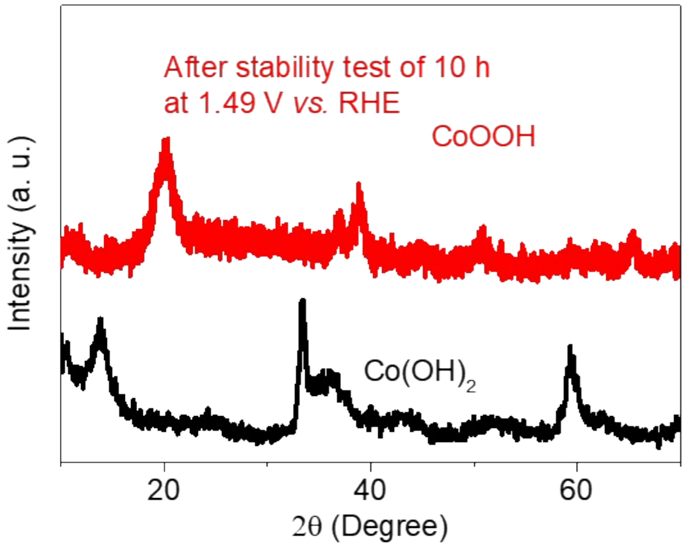

Figure S3. Electrooxidation of $Co(OH)_2$ at 1.49 V *vs.* RHE for 10 h.

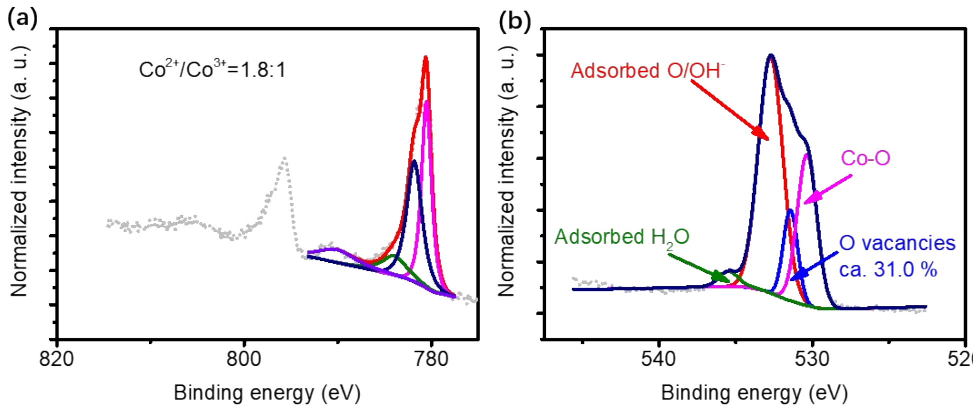

Figure S4. XPS data of Au-Co(OH)$_2$ NSs after use. Co 2p (a) and O 1s fine spectrum.

The Co^{2+}/Co^{3+} ratio is 1.8:1, near to the initial value in as-prepared Au-Co(OH)$_2$ NSs. This result verifies that not all Co cations in Au-Co(OH)$_2$ NSs is participated into OER, which is different with pure Co(OH)$_2$ NSs, indicating Au doping on Co(OH)$_2$ NSs can change the oxidation state of Co cations. The Adsorbed O/OH^- group peaks show increased intensity after use, indicating that electrooxidation activated Co cations become more active with OH^- in alkaline solution, which could promote the electrooxidation of Co at high potential. Adsorbed water molecules occurs on Au-Co(OH)$_2$ NSs surface after use, more likely because water molecules [14]would contribute to the reconstruction of Au-Co(OH)$_2$ NSs in OER. The enhanced Co-O bonding could be resulted by the increased ratio of Co^{3+} in NSs.

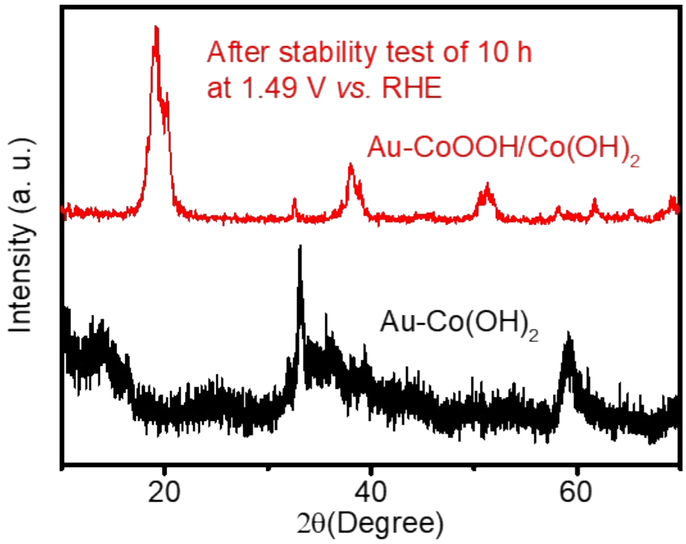

Figure S5. *Ex-situ* XRD of Au-Co(OH)$_2$ NSs after use. The red curve shows that Co(OH)$_2$ is sustained after use, demonstrating the selected electrooxidation process in Au-Co(OH)$_2$ NSs during OER. With the help of *ex-situ* TEM resluts in manuscript, we conclude that the Co surrounded Au is prefrentially oxidized during OER. Meanwhile, this result demonstrates the Au doping can promote the oxidation process of Co^{2+} to Co^{3+}. This promoted formation of Co^{3+} is also proved by XPS data by comparing the ratio of Co^{2+}/Co^{3+}.

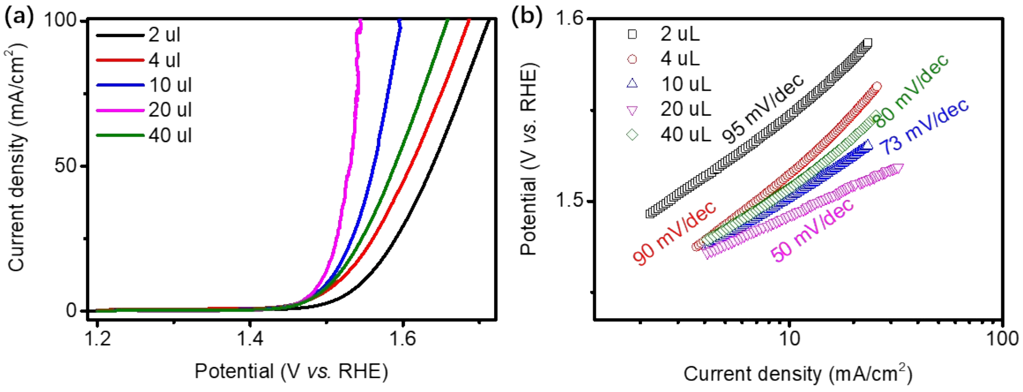

Figure S6. Electrochemical characterization of Au-Co$(OH)_2$ NSs with different Au doping.

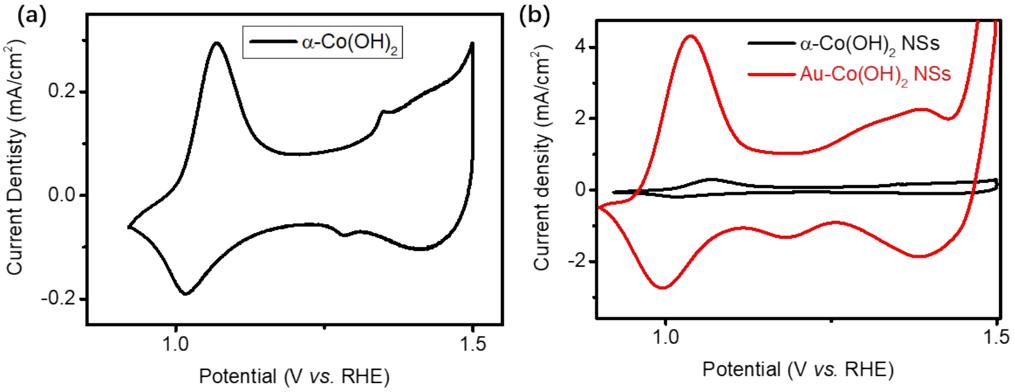

Figure S7. Electrochemical characterization of electrooxidation process of Co^{2+} to Co^{3+} and Co^{3+} to Co^{4+}. Au doping will lead the oxidation peak (Co^{2+}/Co^{3+}) red shift and area increase, as shown in Figure S7b (around 1.1 V).

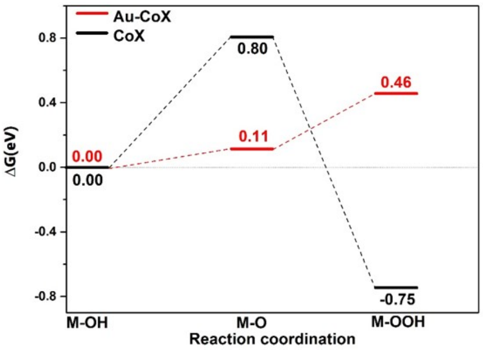

Figure S8. The free energy profiles of high-valence Co cations formation.

Table S2. Average electron numbers of Co, O and H.

number of electrons (e)	Co(OH)2	Au-Co(OH)2
Co	7.91	7.80
O1	7.09	7.02
O2	7.08	7.07
H1	0.46	0.46
H2	0.46	0.42

Here, to illustrate how Au atoms affect the behaviors of Co, we adopt the transition state theory to look inside the reaction mechanism. As shown in Figure S8 and Table S2, after the decoration of Au, $Co(OH)_2$ is facing a smaller barrier changing to CoOOH, in our calculation, the intermediate ought to be CoO_2. Besides, according to Bader Charge Analysis, we got the number of electrons around each atoms. It is clear that Co is exposed to less electrons with Au, which means the Co owns a higher valence.

Free energy calculation:

1.The calculations of Gibbs free energy changes in AEM pathway are as follows:

$$\Delta G_{A1} = \Delta G_{OH^*} - \Delta G_{*} - eU + \Delta G_{pH}$$

$$\Delta G_{A2} = \Delta G_{O^*} - \Delta G_{OH^*} - eU + \Delta G_{pH}$$

$$\Delta G_{A3} = \Delta G_{OOH^*} - \Delta G_{O^*} - eU + \Delta G_{pH}$$

$$\Delta G_{A4} = \Delta G_{O_2^*} - \Delta G_{OOH^*} - eU + \Delta G_{pH}$$

$$\Delta G_{A5} = \Delta G_{*} - \Delta G_{O_2^*} - eU + \Delta G_{pH}$$

2. The calculations of Gibbs free energy changes in LOM pathway are as follows:

$$\Delta G_{L1} = \Delta G_{OO^*} - \Delta G_{OH^*} - eU + \Delta G_{pH}$$

$$\Delta G_{L2} = \Delta G_{OO-OH^*} - \Delta G_{OO^*} - eU + \Delta G_{pH}$$

$$\Delta G_{L3} = \Delta G_{OH-hole^*} - \Delta G_{OO-OH^*} - eU + \Delta G_{pH}$$

$$\Delta G_{L4} = \Delta G_{OH-OH^*} - \Delta G_{OH-hole^*} - eU + \Delta G_{pH}$$

$$\Delta G_{L5} = \Delta G_{OH^*} - \Delta G_{OH-OH^*} - eU + \Delta G_{pH}$$

The analysis is at standard condition (T = 298.15 K, P = 1 bar, pH = 0) and U=0. The Gibbs free energy differences of these intermediates include zero-point energy (ZPE) and entropy according to $\Delta G_i = \Delta E_i + \Delta ZPE_i - T\Delta S_i$, where the energy differences ΔE_i are calculated with respect to H_2O and H_2 (at U = 0 and pH = 0). And the $\Delta G_{pH} = -k_BT \ln(10) \times pH$.

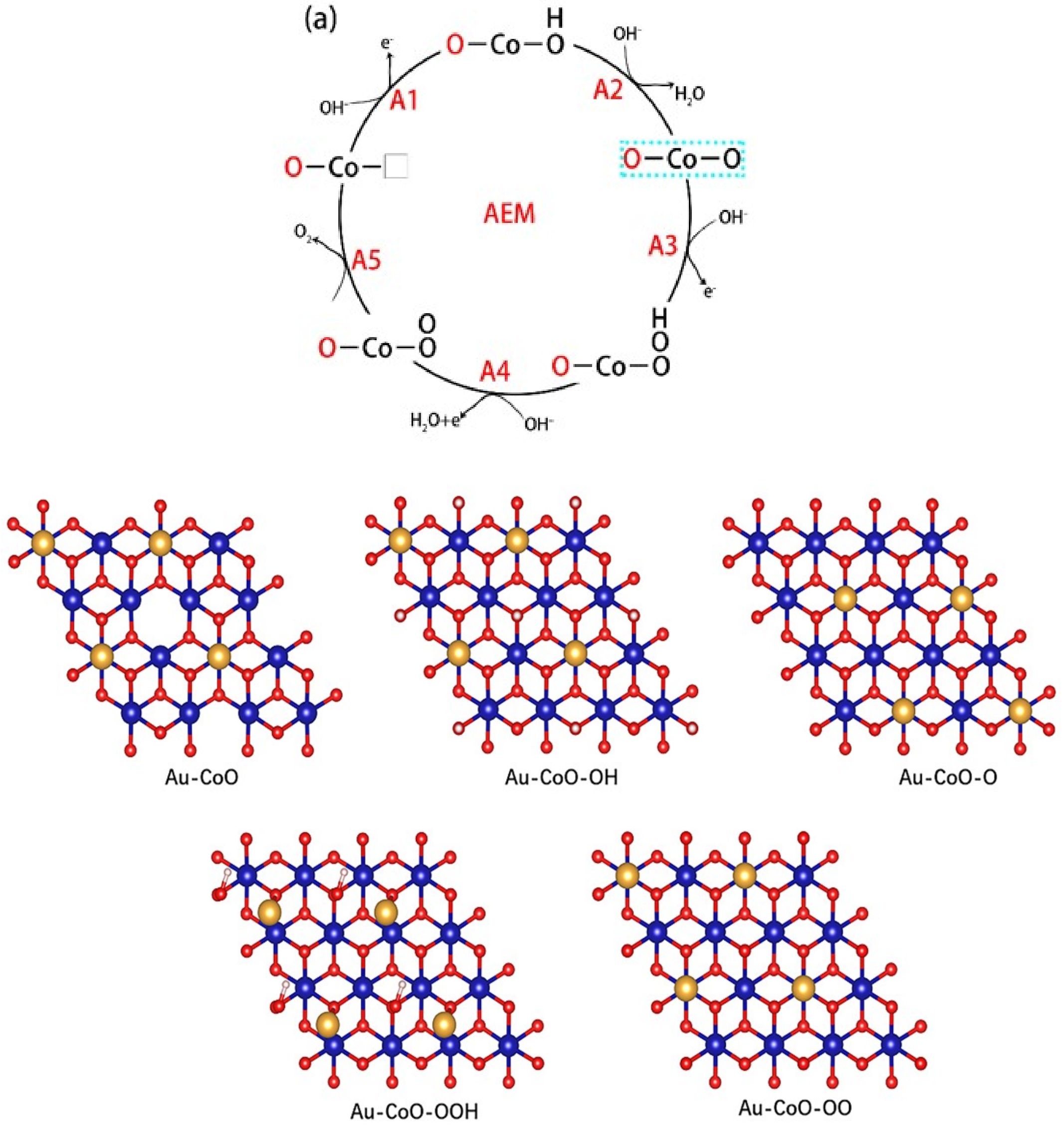

Figure S9. The reaction pathway of AEM and its OER models. Gold balls are Au atoms. Blue balls are Co atoms. Red balls are O atoms. White balls are H atoms.

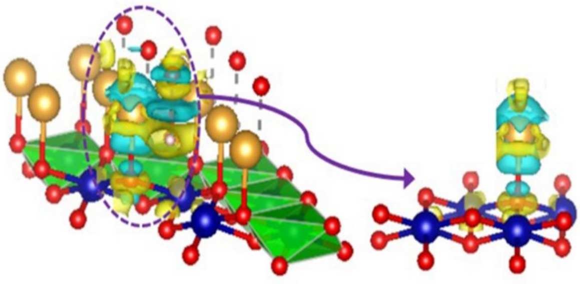

Figure S10. The differential charge densities of Au-$Co(OH)_2$ model with and without

adsorbed Au atom

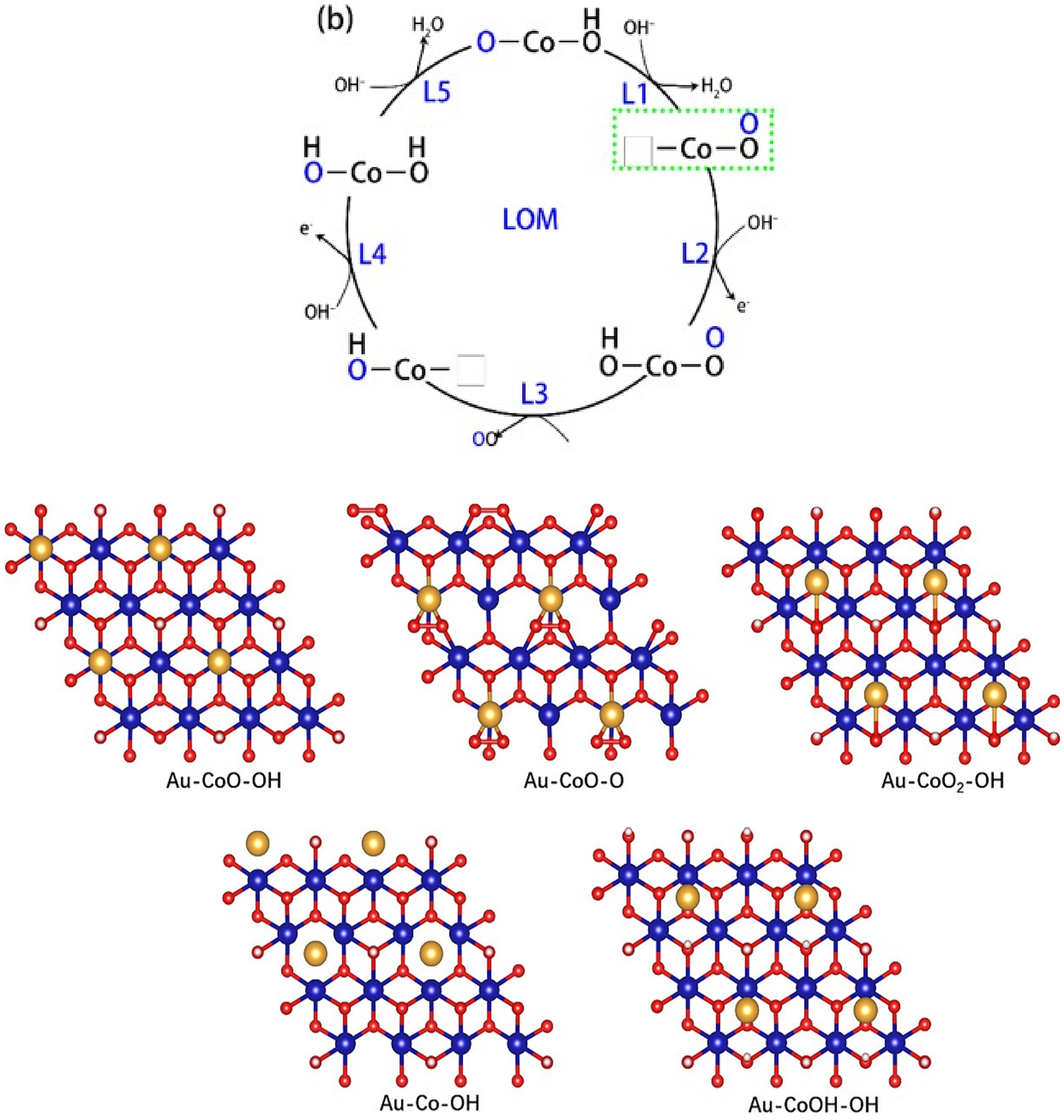

Figure S11. The reaction pathway of LOM and its OER models

Table S3. The calculated zero-point energy and entropy.

	E(eV)	ZPE(eV)	TS(eV)
H_2	-6.88	0.28	0.40
O_2	-9.86	0.1	0.63
H_2O	-14.23	0.57	0.67
*OH		0.37	
*O		0.04	
*OOH		0.43	
*O2		0.15	

References and notes

1. Zhang, J.; Liu, J.; Xi, L.; Yu, Y.; Chen, N.; Sun, S.; Wang, W.; Lange, K. M.; Zhang, B., Single-Atom Au/NiFe Layered Double Hydroxide Electrocatalyst: Probing the Origin of Activity for Oxygen Evolution Reaction. *Journal of the American Chemical Society* **2018,** *140* (11), 3876-3879.
2. Guo, X.; Li, X.; Kou, S.; Yang, X.; Hu, X.; Ling, D.; Yang, J., Plasmon-enhanced electrocatalytic hydrogen/oxygen evolution by Pt/Fe–Au nanorods. *Journal of Materials Chemistry A* **2018,** *6* (17), 7364-7369.
3. Tsai, H.-C.; Vedhanarayanan, B.; Lin, T.-W., Freestanding and Hierarchically Structured Au-Dendrites/3D-Graphene Scaffold Supports Highly Active and Stable Ni3S2 Electrocatalyst toward Overall Water Splitting. *ACS Applied Energy Materials* **2019**.
4. Vassalini, I.; Borgese, L.; Mariz, M.; Polizzi, S.; Aquilanti, G.; Ghigna, P.; Sartorel, A.; Amendola, V.; Alessandri, I., Enhanced Electrocatalytic Oxygen Evolution in Au-Fe Nanoalloys. *Angewandte Chemie* **2017,** *56* (23), 6589-6593.
5. Xu, Y.; Duan, S.; Li, H.; Yang, M.; Wang, S.; Wang, X.; Wang, R., Au/Ni12P5 core/shell single-crystal nanoparticles as oxygen evolution reaction catalyst. *Nano Research* **2017,** *10* (9), 3103-3112.
6. Strickler, A. L.; Escudero-Escribano, M. A.; Jaramillo, T. F., Core-Shell Au@Metal-Oxide Nanoparticle Electrocatalysts for Enhanced Oxygen Evolution. *Nano letters* **2017,** *17* (10), 6040-6046.

7. Hu, E.; Feng, Y.; Nai, J.; Zhao, D.; Hu, Y.; Lou, X. W., Construction of hierarchical Ni–Co–P hollow nanobricks with oriented nanosheets for efficient overall water splitting. *Energy & Environmental Science* **2018,** *11* (4), 872-880.

8. Kwak, I. H.; Im, H. S.; Jang, D. M.; Kim, Y. W.; Park, K.; Lim, Y. R.; Cha, E. H.; Park, J., CoSe(2) and NiSe(2) Nanocrystals as Superior Bifunctional Catalysts for Electrochemical and Photoelectrochemical Water Splitting. *ACS applied materials & interfaces* **2016,** *8* (8), 5327-34.

9. Fang, Z.; Peng, L.; Qian, Y.; Zhang, X.; Xie, Y.; Cha, J. J.; Yu, G., Dual Tuning of Ni-Co-A (A = P, Se, O) Nanosheets by Anion Substitution and Holey Engineering for Efficient Hydrogen Evolution. *Journal of the American Chemical Society* **2018,** *140* (15), 5241-5247.

10. Zhao, X.; Gao, P.; Yan, Y.; Li, X.; Xing, Y.; Li, H.; Peng, Z.; Yang, J.; Zeng, J., Gold atom-decorated CoSe2 nanobelts with engineered active sites for enhanced oxygen evolution. *J. Mater. Chem. A* **2017,** *5* (38), 20202-20207.

11. Zhuang, Z.; Sheng, W.; Yan, Y., Synthesis of Monodispere Au@Co3O4 Core-Shell Nanocrystals and Their Enhanced Catalytic Activity for Oxygen Evolution Reaction. *Advanced Materials* **2014,** *26* (23), 3950-3955.

12. Cai, C.; Han, S.; Caiyang, W.; Zhong, R.; Tang, Y.; Lawrence, M. J.; Wang, Q.; Huang, L.; Liang, Y.; Gu, M., Tracing the Origin of Visible Light Enhanced Oxygen Evolution Reaction. *Advanced Materials Interfaces* **2018**, 1801543.

13. Huang, J.; Chen, J.; Yao, T.; He, J.; Jiang, S.; Sun, Z.; Liu, Q.; Cheng, W.; Hu, F.; Jiang, Y.; Pan, Z.; Wei, S., CoOOH Nanosheets with High Mass Activity for Water Oxidation. *Angewandte Chemie* **2015,** *54* (30), 8722-7.

14. Yeo, B. S.; Bell, A. T., Enhanced Activity of Gold-Supported Cobalt Oxide for the Electrochemical Evolution of Oxygen. *Journal of the American Chemical Society* **2011,** *133* (14), 5587-5593.

1. Zhang, J.; Liu, J.; Xi, L.; Yu, Y.; Chen, N.; Sun, S.; Wang, W.; Lange, K. M.; Zhang, B., Single-Atom Au/NiFe Layered Double Hydroxide Electrocatalyst: Probing the Origin of Activity for Oxygen Evolution Reaction. *Journal of the American Chemical Society* **2018,** *140* (11), 3876-3879.

2. Guo, X.; Li, X.; Kou, S.; Yang, X.; Hu, X.; Ling, D.; Yang, J., Plasmon-enhanced electrocatalytic hydrogen/oxygen evolution by Pt/Fe–Au nanorods. *Journal of Materials Chemistry A* **2018,** *6* (17), 7364-7369.

3. Tsai, H.-C.; Vedhanarayanan, B.; Lin, T.-W., Freestanding and Hierarchically Structured Au-Dendrites/3D-Graphene Scaffold Supports Highly Active and Stable Ni3S2 Electrocatalyst toward Overall Water Splitting. *ACS Applied Energy Materials* **2019**.

4. Vassalini, I.; Borgese, L.; Mariz, M.; Polizzi, S.; Aquilanti, G.; Ghigna, P.; Sartorel, A.; Amendola, V.; Alessandri, I., Enhanced Electrocatalytic Oxygen Evolution in Au-Fe Nanoalloys. *Angewandte Chemie* **2017,** *56* (23), 6589-6593.

5. Xu, Y.; Duan, S.; Li, H.; Yang, M.; Wang, S.; Wang, X.; Wang, R., Au/Ni12P5 core/shell single-crystal nanoparticles as oxygen evolution reaction catalyst. *Nano Research* **2017,** *10* (9), 3103-3112.

6. Strickler, A. L.; Escudero-Escribano, M. A.; Jaramillo, T. F., Core-Shell Au@Metal-Oxide Nanoparticle Electrocatalysts for Enhanced Oxygen Evolution. *Nano letters* **2017,** *17* (10), 6040-6046.

7. Hu, E.; Feng, Y.; Nai, J.; Zhao, D.; Hu, Y.; Lou, X. W., Construction of hierarchical Ni–Co–P hollow nanobricks with oriented nanosheets for efficient overall water splitting. *Energy & Environmental Science* **2018,** *11* (4), 872-880.

8. Kwak, I. H.; Im, H. S.; Jang, D. M.; Kim, Y. W.; Park, K.; Lim, Y. R.; Cha, E. H.; Park, J., CoSe(2) and NiSe(2) Nanocrystals as Superior Bifunctional Catalysts for Electrochemical and

Photoelectrochemical Water Splitting. *ACS applied materials & interfaces* **2016,** *8* (8), 5327-34.
9. Fang, Z.; Peng, L.; Qian, Y.; Zhang, X.; Xie, Y.; Cha, J. J.; Yu, G., Dual Tuning of Ni-Co-A (A = P, Se, O) Nanosheets by Anion Substitution and Holey Engineering for Efficient Hydrogen Evolution. *Journal of the American Chemical Society* **2018,** *140* (15), 5241-5247.
10. Zhao, X.; Gao, P.; Yan, Y.; Li, X.; Xing, Y.; Li, H.; Peng, Z.; Yang, J.; Zeng, J., Gold atom-decorated CoSe2 nanobelts with engineered active sites for enhanced oxygen evolution. *J. Mater. Chem. A* **2017,** *5* (38), 20202-20207.
11. Zhuang, Z.; Sheng, W.; Yan, Y., Synthesis of Monodispere Au@Co3O4 Core-Shell Nanocrystals and Their Enhanced Catalytic Activity for Oxygen Evolution Reaction. *Advanced Materials* **2014,** *26* (23), 3950-3955.
12. Cai, C.; Han, S.; Caiyang, W.; Zhong, R.; Tang, Y.; Lawrence, M. J.; Wang, Q.; Huang, L.; Liang, Y.; Gu, M., Tracing the Origin of Visible Light Enhanced Oxygen Evolution Reaction. *Advanced Materials Interfaces* **2018**, 1801543.
13. Huang, J.; Chen, J.; Yao, T.; He, J.; Jiang, S.; Sun, Z.; Liu, Q.; Cheng, W.; Hu, F.; Jiang, Y.; Pan, Z.; Wei, S., CoOOH Nanosheets with High Mass Activity for Water Oxidation. *Angewandte Chemie* **2015,** *54* (30), 8722-7.
14. Yeo, B. S.; Bell, A. T., Enhanced Activity of Gold-Supported Cobalt Oxide for the Electrochemical Evolution of Oxygen. *Journal of the American Chemical Society* **2011,** *133* (14), 5587-5593.
15. Huang, Z.-F.; Song, J.; Du, Y.; Xi, S.; Dou, S.; Nsanzimana, J. M. V.; Wang, C.; Xu, Z. J.; Wang, X., Chemical and structural origin of lattice oxygen oxidation in Co–Zn oxyhydroxide oxygen evolution electrocatalysts. *Nature Energy* **2019,** *4* (4), 329-338.
